# Evaluation of Biofilm Cultivation Models for Efficacy Testing of Disinfectants against *Salmonella* Typhimurium Biofilms

**DOI:** 10.3390/microorganisms11030761

**Published:** 2023-03-16

**Authors:** Anja M. Richter, Katharina Konrat, Ane M. Osland, Emma Brook, Claire Oastler, Lene K. Vestby, Rebecca J. Gosling, Live L. Nesse, Mardjan Arvand

**Affiliations:** 1 Hospital Hygiene, Infection Prevention and Control, Department Infectious Diseases, Robert Koch Institute, 13353 Berlin, Germany; 2 Department of Analysis and Diagnostics, Norwegian Veterinary Institute, 1433 Ås, Norway; 3 Department of Bacteriology, Animal and Plant Health Agency, Addlestone, Surrey KT15 3NB, UK; 4 Department of Animal Health, Welfare and Food Safety, Norwegian Veterinary Institute, 1433 Ås, Norway; 5 Department of Infectious Diseases, Medical Microbiology and Hygiene, University of Heidelberg, 69120 Heidelberg, Germany

**Keywords:** disinfection efficacy testing, biofilm, *Salmonella*, glutaraldehyde, peracetic acid, biocide

## Abstract

Within the European Union, *Salmonella* is frequently reported in food and feed products. A major route of transmission is upon contact with contaminated surfaces. In nature, bacteria such as *Salmonella* are often encountered in biofilms, where they are protected against antibiotics and disinfectants. Therefore, the removal and inactivation of biofilms is essential to ensure hygienic conditions. Currently, recommendations for disinfectant usage are based on results of efficacy testing against planktonic bacteria. There are no biofilm-specific standards for the efficacy testing of disinfectants against *Salmonella*. Here, we assessed three models for disinfectant efficacy testing on *Salmonella* Typhimurium biofilms. Achievable bacterial counts per biofilm, repeatability, and intra-laboratory reproducibility were analyzed. Biofilms of two *Salmonella* strains were grown on different surfaces and treated with glutaraldehyde or peracetic acid. Disinfectant efficacy was compared with results for planktonic *Salmonella*. All methods resulted in highly repeatable cell numbers per biofilm, with one assay showing variations of less than 1 log_10_ CFU in all experiments for both strains tested. Disinfectant concentrations required to inactivate biofilms were higher compared to planktonic cells. Differences were found between the biofilm methods regarding maximal achievable cell numbers, repeatability, and intra-laboratory reproducibility of results, which may be used to identify the most appropriate method in relation to application context. Developing a standardized protocol for testing disinfectant efficacy on biofilms will help identify conditions that are effective against biofilms.

## 1. Introduction

In the past decade, approximately 90,000 cases of salmonellosis have been reported annually in the European Union (EU)/European Economic Area countries, classifying it as the second most common gastrointestinal infection in this region [1,2]. Case numbers are believed to be much higher due to different reporting strategies, inadequate or lacking microbiological diagnostics, and the often mild and self-limiting course of the disease, where patients do not seek medical care. With 45% of Rapid Alert System for Food and Feed (RASFF) alerts, salmonellosis-inducing non-typhoidal *Salmonella* is the most frequently reported pathogen in food products within EU member states, with the majority being associated with poultry meat and other meat products [3]. Several RASFF alerts have been associated with recurring manufacturers or producers [3], suggesting persistent reservoirs of *Salmonella* within their production lines, most likely within biofilms.

For zoonosis such as salmonellosis, a major route of transmission between animals or within food processing lines is upon contact with contaminations in the environment. In nature, the majority of bacteria are organized in biofilms, which represent microbial aggregates embedded in a self-produced extracellular matrix consisting of protein, polysaccharides, and eDNA. Biofilms can be found to persist as aggregates within suspension as well as adhered to surfaces. Biofilm matrix composition and structure are influenced by environmental conditions such as nutrient and oxygen availability, humidity, temperature, shear forces, and by microbial species present in the biofilm, as well as their individual genetic backgrounds (reviewed in [4,5,6,7,8]). The biofilm matrix serves as a physical and chemical barrier for biocides. In combination with the decreased metabolic activity and enhanced stress tolerance of biofilm cells, biofilm communities show an increased tolerance towards antimicrobials, including disinfectants [6,9,10], compared to planktonic bacteria. In *Salmonella* biofilms, the most common matrix components are amyloid curli fimbriae and cellulose, but other exopolysaccharides, lipopolysaccharides, and surface proteins have been described [11,12,13,14,15]. The biofilm forming ability of individual strains has been observed as an important feature in the persistence of microbial contaminants. For example, good biofilm-forming capabilities have been documented for repeatedly isolated *Salmonella* spp., *Listeria* spp., and *Pseudomonas* spp. from food and feed production lines [16,17,18].

Difficulty in removing biofilms from various surfaces, which are hard to reach, is a major issue in reprocessing medical products, as well as in the entire food production cycle from feed production, farms, abattoirs, and food processing plants [16,19]. Therefore, cleaning and disinfection protocols are routinely used in different settings such as medical, animal husbandry, and food production. The aim is to reduce the microbial burden on surfaces and to prevent the transmission of pathogenic microorganisms from these sources. Glutaraldehyde (GA) and peracetic acid (PAA) are found in a wide range of commercially available disinfectant products for medical, agricultural, and industrial applications. While GA facilitates the cross-linking of macromolecules, PAA increases cell wall permeability and has an oxidizing effect on proteins and polysaccharides [20]. Within the medical, veterinary, and industrial sectors, standardized disinfectant efficacy testing is conducted with planktonic cells in suspension or dried onto a surface [21]. The latter are not organized in multilayer structures and are not embedded in an extracellular matrix, thus being far from the complexity of a biofilm community. Consequently, disinfectant concentrations that prove to be sufficient against planktonic cells often fail to inactivate biofilm-associated cells of the same strain [22,23,24]. In practice, the control of biofilm-associated bacteria with potentially insufficient disinfection concentrations bears the risk of inefficient inactivation, thus leading to the persistence of pathogens and the emergence of tolerance towards disinfectants or other antimicrobials, including antibiotics [25,26]. Therefore, it is crucial to develop and evaluate suitable methods to perform the efficacy testing of disinfectants on biofilm-associated pathogens. 

The aim of this study was to assess three different models for performing the efficacy testing of disinfectants on bacterial biofilms, which have been developed and published by different laboratories [27,28,29]. We evaluated the achievable culturable bacterial counts (colony forming units, CFU) per untreated biofilm, the repeatability of the biofilm cultivation method, and the intra-laboratory reproducibility of each method using *Salmonella* Typhimurium as a model organism. The research groups involved in this study have a focus on pathogen control and disinfection; however, their expertise varies within the medical, veterinary, or agricultural context. Consequently, medical as well as non-medical definitions for successful disinfection were applied for the evaluation of different biofilm models and were compared with the disinfection of cells in suspension. 

## 2. Materials and Methods

### 2.1. Bacterial Strains

To assess disinfectant efficacy against planktonic and biofilm-associated bacteria, *Salmonella enterica* sub. *enterica* serovar Typhimurium reference strains ATCC 14028 and ATCC 13311 were used, thus representing a strong and a weak biofilm performer, respectively. Cells were stored at −80 °C and prior to each experiment recovered in Tryptic Soy Broth (TSB), 5% Sheep Blood Agar (SBA), or Luria Bertani (LB) medium. Unless stated otherwise, cultures for pre-biofilm cultivation and plates for the enumeration of CFU were incubated overnight at 37 °C under aerobic conditions. 

### 2.2. Disinfection and Neutralization Conditions 

For disinfection experiments, bacteria were exposed to PAA (Lerasept^®^ Spezial, Stockmeier Chemie, Bielefeld, Germany) and GA (Protectol^®^ GA 50, BASF, Ludwigshafen, Germany) with contact times of 10 min (PAA) or 30 min (GA), respectively. Following the indicated contact time, PAA was neutralized with 1.65% sodium sulfite in Phosphate-buffered saline (PBS) (0.1 M, pH 7) for 10 s ± 1 s and GA with 20 g/L glycine, 10 g/L Tween 80 in 0.25 M PBS for 5 min ± 10 s. Disinfection and neutralization were performed at 20 ± 1 °C. 

The validation of neutralization and the verification of the absence of toxicity of the respective neutralizers were tested with the highest product test concentration used (for PAA 0.5% and for GA 1%, both *w*/*w*) and for both test strains according to EN 1656:2019 [30]. Disinfectants were diluted with hard water (HW), which was prepared according to EN 1656:2019 [30]. Freshly prepared HW was used within 12 h and had a final pH of 7.0 ± 0.2 when measured at 20 ± 1 °C. 

### 2.3. Definition of Successful Disinfection 

Within the veterinary and food-industrial sector, standardized protocols for disinfectant efficacy testing against bacterial suspensions urge a minimal reduction of 5 log_10_ CFU for successful disinfection (EN 1656, EN 1276), whereas protocols for testing fixed bacteria on non-porous or porous surfaces define successful disinfection as a ≥4 log_10_ reduction of CFU (EN 14349, EN 13697, EN 16437). For the medical sector, both suspension and surface disinfection indicate a ≥5 log_10_ reduction in CFU as a threshold for successful disinfection (EN 13727, EN 14561, EN 16615, EN 17387) (Table 1). In the present study, we considered both target values for the definition of successful disinfection in order to take both medical and non-medical sectors into account. 

### 2.4. Disinfectant Testing in Suspension Assay 

The evaluation of disinfectant efficacy against planktonic *S.* Typhimurium was performed according to the quantitative suspension test EN 1656:2019 [30]. A test suspension of 1.5 × 10^8^ to 5.0 × 10^8^ CFU/mL *S.* Typhimurium was made before serial dilution and plating for enumeration. Briefly, test suspension was mixed with interfering substance (3 g/L Bovine Serum Albumin) to model low-level soiling conditions, at a 1:1 ratio and incubated for 2 min at 20 °C. Then, the mixture was incubated at 20 °C at a ratio of 1:4 with varying concentrations of PAA or GA for the relevant contact time and was neutralized thereafter at 20 °C (1 mL suspension/disinfectant mixture + 1 mL H_2_O + 8 mL neutralizer). Serial dilutions of the test mixture were used to inoculate TSB agar using the pour plate technique and to quantify viable cells (recoverable CFU/mL). Effective disinfection was defined by a minimum of 5 log_10_ reduction (Table 1) in recoverable CFU/mL compared to the untreated test suspension. Experiments were performed three times by one laboratory.

### 2.5. Disinfectant Testing in Biofilm Assay 

For the effective disinfection of biofilms, two target values were considered (≥5 log_10_ and ≥4 log_10_ reduction), defining thresholds for successful disinfection in medical and non-medical sectors, respectively (Table 1). To be able to detect a 4 log_10_ or 5 log_10_ reduction in bacterial counts upon treatment, an initial CFU per mL or per biofilm of ≥10^6^ was defined as a requirement for disinfectant testing. Biofilm cultivation was performed in LB without NaCl, which has been shown to stimulate extracellular matrix production and therefore biofilm formation [31]. Assay-specific limits of detection were applied when no viable cells were observed. Their value depends on the initial volume in which the biofilm was resuspended, the serial dilution, and the total volume that was finally spread on agar plates to enumerate the CFU, and it is therefore calculated from the logarithm of the dilution factor of biofilm cells used for quantification.

#### 2.5.1. Glass Bead Assay (GBA) 

The glass bead assay (GBA) was performed as described previously [23,27]. Briefly, biofilms were cultivated on 4 mm porous glass beads (Sinterglas Pellets, ROBU Glasfilter-Geräte GmbH, Hattert, Germany) in 24-well microplates (one bead per well). Each well was inoculated with 1 mL LB without NaCl containing 10^5^ CFU/mL *S.* Typhimurium. Plates were incubated on an orbital shaker at 100 rpm at 20 °C for 48 h, allowing biofilm cultivation under high shear stress conditions. Thereafter, each bead was carefully dipped in 2 mL sterile H_2_O to remove non-adherent cells and was placed in a 2 mL microcentrifuge tube containing 0.2 mL disinfectant. After incubation for the defined contact time, 1.8 mL of the neutralizing agent was added. Subsequently, bacteria were sonicated in an ultrasonic bath (BactoSonic^®^, Bandelin, Berlin, Germany) at 40 kHz for 10 min using 200 W_eff_ to detach biofilm cells from the bead surface and were quantified by serial dilution in neutralizing agent. A total of 5 µL of every dilution as well as 1000 µL of the undiluted suspensions and the first dilution were plated on TSB agar. The controls were treated with HW instead of disinfectant. Considering the volumes used for biofilm resuspension and CFU quantification in this assay, the dilution factor was 8.88 (set to 10 here), resulting in a limit of detection of 10^1^ CFU/mL (or 1 log_10_). Experiments were performed three times by one technician with three technical replicates each.

#### 2.5.2. PVC Coupon Assay (PCA)

In the PVC coupon assay (PCA), biofilms were grown on Polyvinyl carbonate (PVC) coupons as described previously [28], with the following modifications. A standard inoculum was prepared from the overnight SBA plates by aseptically inoculating LB broth without NaCl until the cell suspension reached 1 McFarland (~3 × 10^8^ CFU/mL), using a suspension turbidity meter. A total of 1.5 mL of the bacterial suspension was added into a 12-well microtiter plate, with sterile LB broth without NaCl as a negative control. Sterile PVC coupons (10 × 20 × 0.6 mm; Pegen Industries Inc., Stittsville, ON, Canada) were placed in the wells so that only the bottom half of the coupon was submerged in medium. Microtiter plates underwent static incubation for 48 h at 20 °C to allow biofilm growth. After incubation, coupons were washed in three consecutive 9 mL sterile saline solutions with light agitation to remove non-adherent bacteria and left to dry at room temperature. For disinfectant efficacy testing, coupons were submerged in 10 mL disinfectant or HW (control) for the specified contact time, before being transferred to 10 mL of the appropriate neutralizing agent. After at least 5 min of neutralization, coupons were transferred to 5 mL of sterile saline together with 30 glass beads (5 mm soda lime glass beads (Z265942; Sigma-Aldrich, Saint Louise, MI, USA) and were vortexed at the lowest speed for 2 min to allow biofilm detachment, before an additional 5 mL saline was added. Suspensions underwent 10-fold serial dilution and 100 µL spread-plated on SBA plates to allow the enumeration of bacteria. Considering the volumes used for biofilm resuspension and CFU quantification in this assay, the dilution factor was 100, resulting in a limit of detection of 10^2^ CFU/biofilm (or 2 log_10_). Experiments were performed three times by one technician with two technical replicates each.

#### 2.5.3. Stainless Steel Coupon Assay (SSCA) 

Biofilms on stainless-steel coupons (Stainless Steel Coupon Assay, SSCA) were cultivated as described in [32] with minor modifications. Plate-grown *S.* Typhimurium was resuspended in 5 mL LB and adjusted to an optical density of 1 McFarland. Then, 0.5 mL cell suspension was added into 10 mL LB without NaCl, and cells were incubated in sterile centrifuge tubes together with an autoclaved stainless-steel coupon of 75 × 24 × 1 mm (Stainless steel AISI304, 2B Olaf Johansens Eftf. A/S, Oslo, Norway). The coupon was half-submerged in medium and incubated under static conditions at 20 °C for 48 h. Thereafter, the coupon was rinsed three times with 40 mL of sterile saline to remove non-adherent cells and was transferred to a tube with 10 mL of disinfectant (or saline for controls) for the indicated contact time. The coupon was then moved to a tube with an appropriate neutralizing agent before it was rinsed again and added to a tube containing 5 mL of saline and 20–30 solid glass beads (2 mm, Avantor, VWR collection, Oslo, Norway). Here, visible biofilm was scraped off both sides of the coupon with a sterile cell scraper (1.8 cm BD Falcon, Bedford, MA, USA). The coupon was discarded and the tube was vortexed for one minute to disperse cell aggregates. Serial dilutions were performed to determine the viable CFU, and 5 µL was plated from each dilution onto blood agar plates using a multi-channel pipette. Considering the volumes used for biofilm resuspension and CFU quantification in this assay, the dilution factor was 1000, resulting in a limit of detection of 10^3^ CFU/biofilm (or 3 log_10_). Experiments were performed three times by one technician with two technical replicates each.

### 2.6. Determination of Repeatability and Reproducibility 

Repeatability was described as the variation between the results of repeated experiments performed by the same person in the same laboratory under the same conditions. For each biofilm model, six independent biofilm experiments (three experiments for PAA and three experiments for GA) were performed by one technician to assess the CFU count of untreated (=control) biofilms with a minimum of two technical replicates per experiment. Biofilm cells were quantified and displayed in CFU/mL (GBA) or in CFU/biofilm (PCA and SSCA). For each biofilm model, repeatability was assigned when the maximal variation of the mean CFU/mL (or biofilm) between individual experiments was within 1 log_10_. In addition, an absolute minimum of 10^6^ CFU/mL (or biofilm) was desired to guarantee sufficiently high initial values for subsequent disinfection tests. CFU/mL (or biofilm) below 10^6^ were considered insufficient for further disinfection experiments. 

Reproducibility was described as the variation between the test results in one laboratory, when the same experiment was performed by two different technicians. For this task, the experiments for the efficacy testing of PAA and GA on *S.* Typhimurium ATCC 14028 were conducted independently by two different technicians in all three participating laboratories. In each laboratory, each technician conducted three independent experiments with at least two technical replicates per disinfectant concentration.

### 2.7. Statistical Analysis 

Reduction in CFU was calculated as follows: First, for each experiment, the mean CFU/mL or biofilm of untreated control suspensions or biofilms was calculated (mean_control_). Then, CFU/mL or the biofilm of samples treated with disinfectant were subtracted from mean_control_ to determine the CFU reduction of individual technical replicates (two or three depending on the biofilm model used). Finally, the mean of those individual technical replicates was calculated, resulting in one mean_reduction_ value for each experimental condition (=GA or PAA concentration, respectively) per experiment. The final bar charts represent the mean reduction values of three or six experiments given in CFU in log_10_. Statistical analysis and data visualization were performed using Prism 9.0 GraphPad Software (Version 9.1.0, La Jolla, CA, USA). The Mann-Whitney-U test was performed to evaluate intra-laboratory reproducibility, assuming significant differences between the two technicians for a *p* value of <0.05.

## 3. Results

### 3.1. Disinfection of Planktonic Salmonella in Suspension 

**Figure 1:**
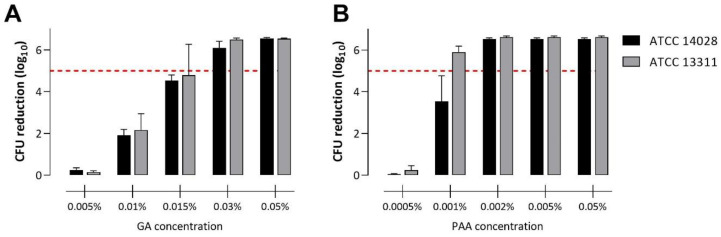
The efficacy of glutaraldehyde (GA) (**A**) and peracetic acid (PAA) (**B**) against planktonic cell suspensions of *S.* Typhimurium ATCC 14028 and ATCC 13311 evaluated with the quantitative suspension assay according to EN 1656. Bars represent mean log_10_ reduction in CFU after GA or PAA treatment compared to the CFU of untreated control suspensions. Dashed lines indicate a 5 log_10_ reduction in CFU and therefore successful disinfection.

Previous studies demonstrated the enhanced tolerance of biofilm-embedded bacteria towards antimicrobial treatment, including disinfection, compared with planktonic bacteria. To compare the disinfectant tolerance of *S.* Typhimurium biofilms with those of planktonic cells, we first evaluated PAA and GA efficacy against cell suspension according to the EN 1656 standard. The successful disinfection (minimum 5 log_10_ reduction in recoverable CFU/mL) of both reference strains with GA was achieved with a concentration of 0.03% (*w*/*w*) at a 30 min contact time (Figure 1A). Using PAA, successful disinfection was achieved with a concentration of 0.001% and 0.002% (*w*/*w*) at a 10 min contact time for *S.* Typhimurium ATCC 13311 and ATCC 14028, respectively (Figure 1B). 

### 3.2. Cultivation of Salmonella Biofilms

#### 3.2.1. Achievable CFU in Untreated Biofilms 

**Figure 2:**
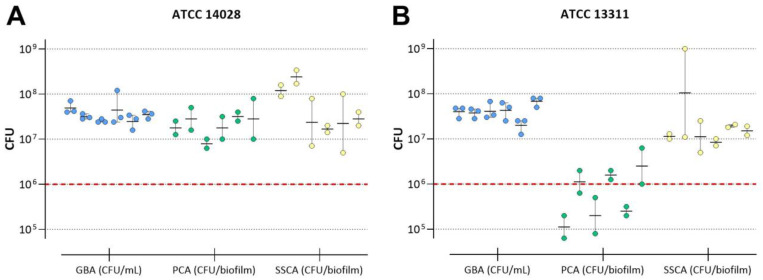
Recovered numbers of viable cells of untreated ATCC 14028 (**A**) and ATCC 13311 (**B**) biofilms given in CFU/mL (GBA, blue) or CFU/biofilm (PCA, green, and SSCA, yellow), respectively. Presented here are the mean CFU/mL or biofilm (horizontal lines) as well as individual values of at least two technical replicates of six experiments performed by the same technician. Colored bullets represent individual biofilm replicates. Dashed red lines indicate the desired minimal cell number of 10^6^ CFU.

In the first step, we determined the achievable average CFU per untreated biofilm for each biofilm model tested. To detect a 4 log_10_ or 5 log_10_ reduction in bacterial counts upon treatment, an initial CFU per mL (GBA) or per biofilm (PCA and SSCA) of ≥10^6^ was defined as a requirement for disinfectant testing. For *S.* Typhimurium ATCC 14028, the minimum requirement of cell numbers in untreated control biofilms was achieved using all three methods (Figure 2A). In contrast, the cultivation of *S.* Typhimurium ATCC 13311 biofilms on PVC coupons resulted in CFU below the desired minimum of 10^6^/biofilm, while cell numbers within the GBA and SSCA were sufficiently high (Figure 2B).

#### 3.2.2. Repeatability of CFU in Untreated Biofilms 

A maximum variation of 1 log_10_ in mean CFU per mL (GBA) or biofilm (PCA and SSCA) was assigned a prerequisite for repeatability.Figure 2 displays the mean CFU of six independent experiments performed by one technician with *S.* Typhimurium ATCC 14028 (A) and ATCC 13311 (B) with at least two individual technical replicates each. For the GBA model, the maximal variation of mean CFU/mL between all six experiments performed with ATCC 14028 as well as ATCC 13311 was ≤1 log_10_ (i.e., 0.3 and 0.53, respectively). For the PCA model, the maximum variation of mean CFU/biofilm was below 1 log_10_ for ATCC 14028 (0.6) but not for ATCC 13311 (1.35). For the SSCA method, higher variations were detected with both strains tested (ATCC 14028: 1.15 and ATCC 13311: 1.09). 

### 3.3. Disinfectant Efficacy Testing of Biofilms 

We next tested the efficacy of GA and PAA to inactivate *Salmonella* when organized in biofilms. As described in detail in the Materials and Methods section, we considered two CFU reduction values, ≥4 log_10_ and ≥5 log_10_, as indicators of the successful disinfection of biofilms in non-medical and medical settings, respectively. Additionally, note the different GA and PAA concentration ranges that were used in the different assays and the assay-specific detection limit (1, 2, and 3 log_10_ for GBA, PCA, and SSCA, respectively), which results in different maximum attainable CFU reduction values in the respective assay.

#### 3.3.1. Disinfection with Glutaraldehyde

**Figure 3:**
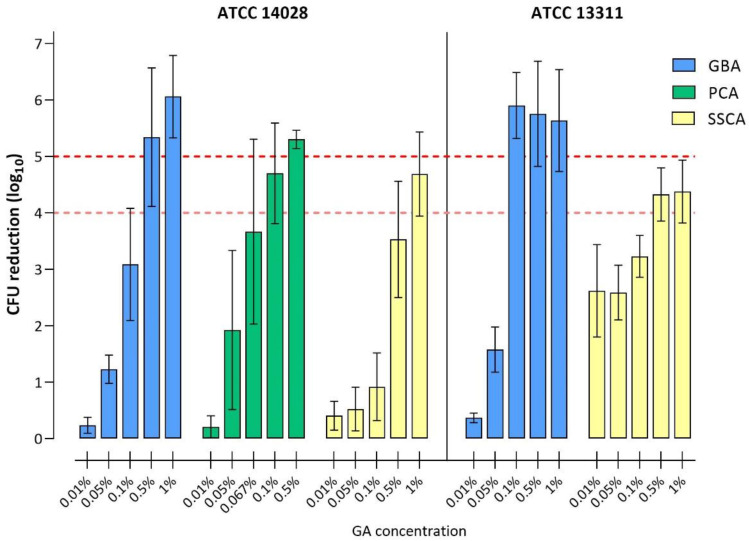
The reduction of viable cells in *S.* Typhimurium ATCC 14028 and ATCC 13311 biofilms following disinfection with GA. Bars represent log_10_ reduction in CFU after GA treatment compared to the CFU of untreated control biofilms. Red dashed lines highlight the 4 log_10_ and 5 log_10_ CFU reductions, which indicate successful disinfection. GBA (blue), PCA (green), and SSCA (yellow). Bars represent the mean with the standard deviation of three (ATCC 13311) or six (ATCC 14028) individual experiments, with at least two biological replicates per experiment. Note the different GA concentration ranges in the different assays as well as the assay-specific detection limits (1, 2, and 3 log_10_ for GBA, PCA, and SSCA, respectively).

The results of efficacy testing using GA are presented in Figure 3 and Table 2. For the GBA and SSCA methods, the GA concentrations tested ranged from 0.01% to 1%, while for the PCA method, the highest GA concentration tested was 0.5%. *S.* Typhimurium ATCC 14028 was subjected to efficacy testing in all three methods, while the ATCC 13311 strain was only assessed with the GBA and the SSCA method, since, in the PCA method, minimal CFU counts of untreated biofilms were not consistently above 10^6^ CFU/biofilm. 

For the ATCC 14028 strain, a 5 log_10_ CFU reduction upon treatment with GA was achieved at 0.5% for both the GBA and PCA models. For the SSCA model, a ≥5 log_10_ CFU reduction could not be determined due to the assay-specific detection limit of 3 log_10_, which resulted in a limited range for determining CFU reduction. For the same strain, a 4 log_10_ reduction was achieved with GA concentrations of 0.5%, 0.1%, and 1% in the GBA, PCA, and SSCA methods, respectively.

For the ATCC 13311 strain, a 5 log_10_ CFU reduction upon treatment with GA was achieved using the GBA model at concentrations of 0.1%. Similar to the ATCC 14028 strain, a ≥5 log_10_ CFU reduction could not be determined using the SSCA model. GA concentrations of 0.1% (GBA) and 0.5% (SSCA) resulted in a 4 log_10_ CFU reduction. Taken together, the GA concentration necessary for the disinfection of biofilms of both *Salmonella* strains was markedly higher than the concentration that is effective for the disinfection (5 log_10_ reduction) of planktonic cells in suspension, i.e., 0.03% for both ATCC 14028 and ATCC 13311. On a strain-specific level, ATCC 14028 biofilms showed a higher tolerance against GA compared to ATCC 13311 biofilms. 

#### 3.3.2. Disinfection with Peracetic Acid

**Figure 4:**
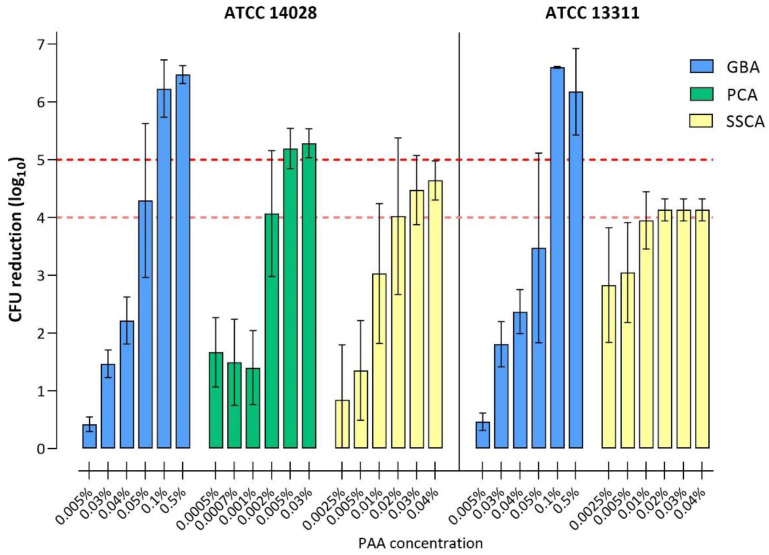
The reduction of viable cells in *S. Typhimurium* ATCC 14028 and ATCC 13311 biofilms following disinfection with PAA. Bars represent log_10_ reduction in CFU after PAA treatment compared to the CFU of untreated control biofilms. Red dashed lines highlight 4 log_10_ and 5 log_10_ CFU reduction, which indicate successful disinfection. GBA (blue), PCA (green), and SSCA (yellow). Bars represent the mean with a standard deviation of three (ATCC 13311) or six (ATCC 14028) individual experiments with at least two biological replicates per experiment. Note the different PAA concentration ranges in the different assays as well as the assay-specific detection limits (1, 2 and 3 log_10_ for GBA, PCA and SSCA, respectively).

Efficacy testing results obtained with PAA are shown in Figure 4 and Table 3. In the GBA method, the PAA concentrations tested ranged from 0.005% to 0.5%, in the PCA method from 0.0005% to 0.03%, and in the SSCA method from 0.0025% to 0.04%. *S.* Typhimurium ATCC 14028 was tested using all three methods, while ATCC 13311 was only tested with GBA and SSCA models. 

For ATCC 14028, a 5 log_10_ CFU reduction was achieved using the GBA and PCA with PAA concentrations of 0.1% and 0.005%, respectively, while for the SSCA, the successful PAA concentration for a 5 log_10_ CFU reduction could not be determined (see GA disinfection). Accordingly, a 4 log_10_ reduction was observed at PAA concentrations of 0.05% (GBA), 0.002% (PCA), and 0.02% (SSCA). 

For ATCC 13311, a 5 log_10_ reduction was achieved in the GBA model with PAA concentrations of 0.1%, yet it could not be determined for the SSCA model. A 4 log_10_ reduction was achieved at 0.1% (GBA) and 0.02% PAA (SSCA). 

In summary, biofilms grown and tested with the GBA and SSCA methods were generally more tolerant to PAA compared to their planktonic counterparts, for which a 5 log_10_ CFU reduction was achieved at 0.002% and 0.001% for ATCC 14028 and ATCC 13311, respectively (Figure 1). In contrast, ATCC 14028 biofilms grown and tested with the PCA method were, in comparison with the GBA and SSCA methods, substantially more susceptible to PAA (4 log_10_ with 0.002%; 5 log_10_ with 0.005%) and were near equivalent to the results obtained for planktonic cells (compare Figure 1).

In addition, the dose-dependent efficacy of both disinfectants, PAA and GA, was shown for all three biofilm disinfection assays.

### 3.4. Intra-Laboratory Reproducibility 

**Figure 5:**
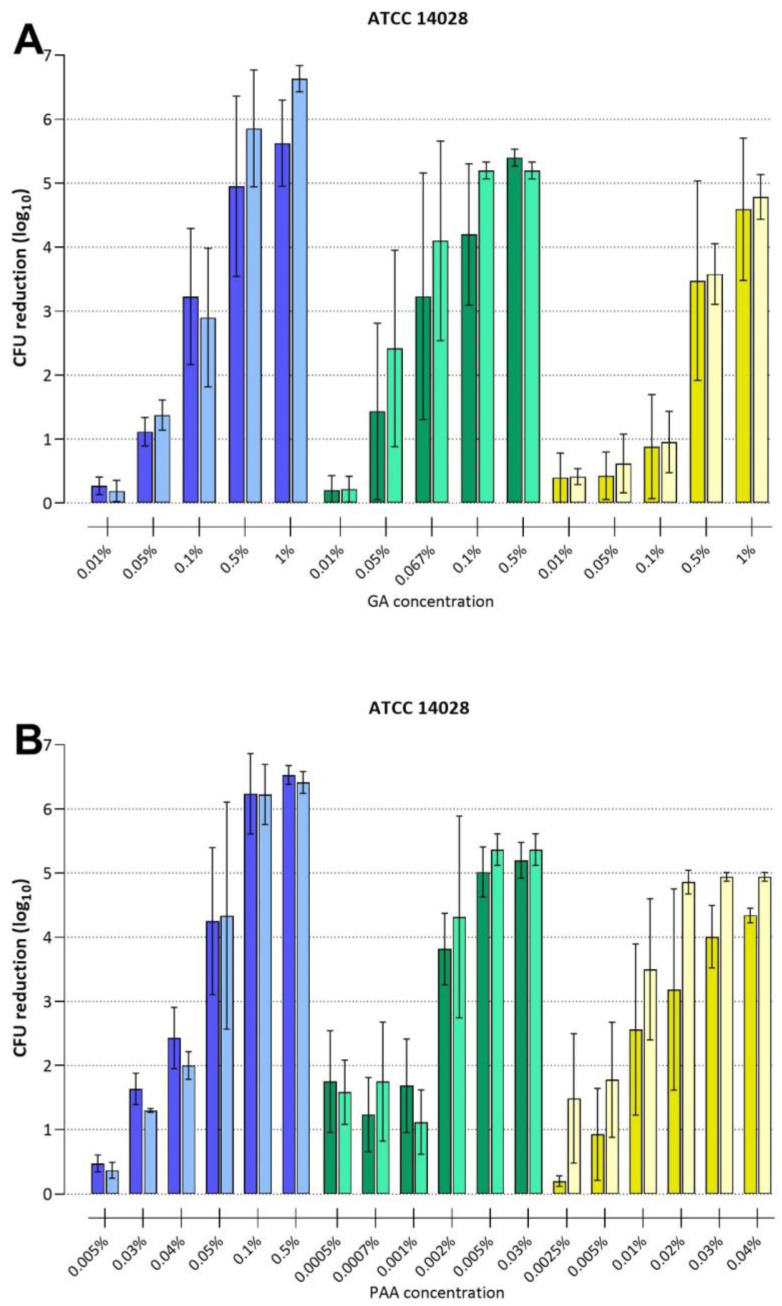
The intra-laboratory reproducibility testing of *S.* Typhimurium ATCC 14028 biofilm disinfection with GA (**A**) or PAA (**B**). The color scheme is identical with previous figures (GBA: blue, PCA: green, and SSCA: yellow), while bright and dark bars indicate results from two different technicians. Bars represent the mean with a standard deviation of three individual experiments performed by the same technician with at least two biological replicates per experiment. Note the variations in GA and PAA concentrations used in all three assays as well as assay-specific detection limits (1, 2, and 3 log_10_ for GBA, PCA, and SSCA, respectively).

Reproducibility is considered a key factor for putative candidates for standardized protocols. To assess the intra-laboratory reproducibility of the different methods used in this study, each biofilm disinfection assay was also performed by a second technician with three experiments and at least two technical replicates each. The results of the efficacy testing experiments for GA and PAA for the ATCC 14028 strain are displayed in Figure 5. 

A comparison of the results of the two technicians for each of the biofilm models was performed using the Mann-Whitney-U test, assuming independent samples and their non-parametric distribution. We did not find any significant differences between the results of the two technicians for any of the biofilm testing models, suggesting that all models warrant a high intra-laboratory reproducibility. 

## 4. Discussion

Within their natural habitats, bacteria are usually organized in multi-layered biofilms, in which individual cells are often more resistant towards harsh environmental conditions such as cleaning and disinfection procedures [6,23,33,34,35]. However, current standards for the evaluation of disinfectants, and therefore recommended user concentrations, are based on efficacy testing against planktonic bacteria either in suspension or fixed to a surface, rather than against biofilms. The present study compared three methods, which allow the cultivation of repeatable and intra-laboratory reproducible *S.* Typhimurium biofilms and their usage for the evaluation of disinfectant efficacy against biofilm-embedded bacteria. All three methods enable the formation of *S.* Typhimurium biofilms on an abiotic surface (glass, PVC, stainless-steel), their treatment with disinfectants, and the further quantification of viable cells. 

For medical instruments and non-porous surfaces, a successful disinfection is defined as a ≥5 log_10_ reduction in viable cells (EN 14561, EN 16615, EN 17387), while in the veterinary area and food industry, the minimal limit is a ≥4 log_10_ reduction (EN 14349, EN 13697) [21]. Depending on the assay and strain used, biofilm disinfection (a ≥5 log_10_ reduction) was achieved with GA concentrations of 0.1 to 0.5% and PAA concentrations of 0.005% to 0.1%. Meanwhile, for a ≥5 log_10_ reduction in the CFU of planktonic cells, lower concentrations proved to be sufficient (0.03% GA and 0.001–0.002% PAA, respectively). Here, a 4 log_10_ or 5 log_10_ reduction of biofilm-embedded cells required higher concentrations of PAA and GA compared to planktonic cells, demonstrating the increased tolerance of bacterial biofilms against disinfectants, as well as the successful formation of such tolerant communities in all three biofilm models. 

If the goal was to detect a CFU reduction of ≥5 log_10_ after exposure to disinfectants, this could only be achieved using GBA and PCA methods, while detecting a reduction of ≥4 log_10_ in CFU was achieved with all three methods. Achieved reductions in viable cells (given in log_10_) depend not only on the tolerance of bacteria but also on specifications of the model used, especially the detection limit of the assay. Here, the detection limit of the GBA, PCA, and SSCA was 1, 2, or 3 log_10_, respectively, depending on the volume used for biofilm dispersal and serial dilutions. Assuming a theoretical mean of 10^7^ CFU before PAA or GA treatment, maximum possible CFU reduction numbers are 6 log_10_, 5 log_10_, and 4 log_10_ (GBA, PCA, and SSCA, respectively), thus resulting in an underestimation of disinfectant efficacy in some assays [36].

The concentrations of GA and PAA identified in this study to be effective for disinfecting *Salmonella* biofilms are in line with results of previous studies that showed a similar concentration range that was effective against biofilms of other pathogens, e.g., *Staphylococcus aureus* [37], *Pseudomonas aeruginosa* [27,37], *Klebsiella pneumoniae* [23], as well as *Salmonella* spp. [38], demonstrating the reliability of the results obtained in this study. It is noteworthy that the disinfectant concentration that proved effective for disinfecting *Salmonella* biofilms was always higher than the concentration required to disinfect their planktonic counterparts. Since the recommended user concentrations for disinfectants are currently based on efficacy test results on planktonic bacteria, our results suggest that these concentrations may not warrant the reliable disinfection of biofilm-associated bacteria. This finding is in line with the observation that biofilms can be often found in situations where cleaning and disinfection measures are inadequate, e.g., in the context of foodborne outbreaks.

*S.* Typhimurium strains with high biofilm formation capacities (ATCC 14028) reached a sufficiently high and consistent number of cells, allowing for further disinfection testing. However, when cultivated on PVC coupons, strains with low biofilm formation capacities (ATCC 13311) may not meet those requirements under the conditions tested, highlighting the importance of the selected test strains. Although previous studies have demonstrated *Salmonella* biofilm formation on stainless steel, glass, and PVC, a strong attachment to the latter was not detected for *Salmonella* spp. isolated from raw poultry [39,40], regardless of incubation time and temperature. To ensure flexibility when choosing the surface material used for biofilm formation, our results suggest including strong biofilm performers as reference strains in standardized methods for testing biofilm disinfection. In addition, previous studies suggest the production of amyloid curli fibers and cellulose in macrocolony biofilms of ATCC 14028 [41], while ATCC 13311 macro-colony biofilms suggest the lack of cellulose. In our study, ATCC 14028 biofilms were more tolerant towards GA compared to ATCC 13311, suggesting that curli and/or cellulose might contribute to altered biofilm composition and to increased tolerance to GA within all biofilm methods tested. 

With regard to the most suitable surface material, e.g., stainless steel, glass, or polymers (PVC, PP, PS), used to study the biofilm formation of *Salmonella* or other food-borne pathogens, previous studies are rather inconsistent. They have shown high variations in strain specificity regarding cell adhesion to individual surface materials [40,42,43,44,45,46,47]. All three assays evaluated here are adaptable regarding the surface materials, provided that carrier devices with defined surface characteristics are used. Thus, disinfectant efficacy against biofilms can be tested on multiple materials relevant within the industrial, agricultural, and veterinary sectors. 

In addition to different carrier materials, the described assays also differ in the application of shear forces during biofilm development. In the GBA, turbid flow and therefore high shear forces were applied during biofilm formation, whereas cells on stainless-steel or PVC coupons grew under static conditions. Fluid dynamics and therefore shear forces during the biofilm formation process have stimulating effects on matrix production and were observed to increase cell density, biomass, and general biofilm stability, affecting the disinfectant penetration into the biofilm [48,49,50,51]. Moreover, decreased biofilm stability may result in the dispersal of loosely attached cell aggregates from the substrate and consequently higher variations of recoverable CFU. Although all three methods have proven their value for reproducible disinfectant efficacy testing on biofilms, the method with shear forces (GBA) resulted in the most robust biofilms with respect to low variations in achievable cell numbers (Figure 2) and might therefore be a candidate to model the worst-case scenarios of biofilm contamination. However, regardless of shear forces applied, a method with a low variation in cell numbers (and thus high repeatability) is advantageous. 

Established biofilm cultivation methods such as the CDC biofilm reactor [50,52], the Drip-Flow reactor [53], or continuous flow cells [54] allow for the testing of a whole range of surface materials, the prolonged cultivation of mature biofilms, and their detailed three-dimensional microscopic visualization, and they can exploit their full potential in biofilm research. However, they require advanced technical equipment and training and are unpracticable for the high-throughput screening of multiple organisms at a time. In contrast, the assays described here proved to be feasible with common laboratory equipment, are adaptable regarding test organism and carrier surface material, and they are scalable to the desired number of test conditions. In addition, they allow the cultivation of biofilms with consistent quality regarding cell numbers—a crucial condition for standardized protocols on efficacy testing. 

The limitations of this study are the small number of technical replicates per experiment and per disinfectant concentration used, limiting the applicable techniques for detailed statistical analysis. However, the number of replicates was in agreement with the current international standards for disinfectant efficacy testing [21,30] and therefore resembles realistic conditions of disinfectant efficacy testing. We found different detection limits of the individual assays, which may lead to the underestimation of the efficacy of disinfectants. If possible, this should be improved in future studies. 

Further steps towards the development of a standardized protocol for biofilm disinfection testing should involve a wider range of tested strains with more variable biofilm formation capacities, for example, wild-type isolates. This will help to identify more suitable test strains, as domesticated laboratory strains often forfeit their ability to form persistent biofilms and might not be suitable for disinfectant evaluation in biofilms [55]. In addition, inter-laboratory reproducibility testing as well as the assessment of feasibility regarding the material–cost ratio, high-throughput screening, and user-friendliness will give further insights into the practicability and comparability of the described methods and to assess their potential as candidates for standardization. Besides the reliable evaluation of disinfectant efficacy, such a standardized protocol for biofilm cultivation and disinfectant efficacy testing is a promising tool for future studies focusing on biofilm-specific tolerance mechanisms and antimicrobial penetration into surface-attached microbial communities. 

## 5. Conclusions

This study aimed to assess different protocols for the cultivation of bacterial biofilms suitable for the quantitative evaluation of disinfectant efficacy against *S.* Typhimurium and to identify suitable candidates for standardization. We found some differences between the tested methods, which can be used to identify the most suitable method in relation to the application context for individual disinfectants. The development of a standardized efficacy testing protocol for biofilms will help to raise awareness for biofilm-associated contamination, as well as to define disinfection conditions that are effective against preexisting biofilms.

## Figures and Tables

**Table 1:** Standardized protocols for the evaluation of disinfectant efficacy within the medical, veterinary, and industrial sectors. Given are the EN standards, test substances (suspension, carrier, surface), and minimal CFU reduction values necessary for successful disinfection [21].

	Veterinary Area	Food, Industrial, Domestic, and Institutional Area	Medical Area
Phase 2, Step 1	Quantitative suspension test EN 1656:2019 (≥5 log_10_)	Quantitative suspension test EN 1276:2019 (≥5 log_10_)	Quantitative suspension test EN 13727:2012+A2:2015 (≥5 log_10_)
Phase 2, Step 2	Quantitative non-porous surface test EN 14349:2012 (≥4 log_10_) Quantitative porous surface test EN 16437:2014+A1:2019 (≥4 log_10_)	Quantitative non-porous surface test EN 13697:2015+A1:2019 (≥4 log_10_)	Quantitative carrier test EN 14561:2006 (instruments, ≥5 log_10_) Quantitative non-porous surface test EN 16615:2015 (surfaces w/mechanical action, ≥5 log_10_) Quantitative non-porous surface test EN 17387:2021 (surfaces w/o mechanical action, ≥5 log_10_)

**Table 2:** Effective GA concentrations for biofilm disinfection using the GBA, PCA, and SSCA models.

Glutaraldehyde	GBA		PCA		SSCA	
CFU Reduction	ATCC 14028	ATCC 13311	ATCC 14028	ATCC 13311	ATCC 14028	ATCC 13311
≥4 log_10_	0.5%	0.1%	0.1%	-	1%	0.5%
≥5 log_10_	0.5%	0.1%	0.5%	-	n.a. *	n.a. *

* not available: Calculation not possible due to assay-specific limit of detection.

**Table 3:** Effective PAA concentration for biofilm disinfection using GBA, PCA, and SSCA models.

Peracetic Acid	GBA		PCA		SSCA	
CFU Reduction	ATCC 14028	ATCC 13311	ATCC 14028	ATCC 13311	ATCC 14028	ATCC 13311
≥4 log_10_	0.05%	0.1%	0.002%	-	0.02%	0.02%
≥5 log_10_	0.1%	0.1%	0.005%	-	n.a. *	n.a. *

* not available: Calculation not possible due to assay-specific limit of detection.

## Data Availability

The data presented in this study are available on request from the corresponding authors.
